# Iatrogenic Spinal Subdural Extra-Arachnoid Hygroma Following Uncomplicated Lumbar Decompression

**DOI:** 10.7759/cureus.1171

**Published:** 2017-04-17

**Authors:** Benjamin D Elder, Wataru Ishida, Rory C Goodwin, Ali Bydon

**Affiliations:** 1 Department of Neurosurgery, The Johns Hopkins University School of Medicine; 2 Neurosurgery, Columbia University Department of Neurosurgery

**Keywords:** arachnoid cyst, durotomy, iatrogenic, laminectomy, posterior decompression, spinal subdural extra-arachnoid hygroma

## Abstract

Intradural spinal arachnoid cysts (ISACs) have been reported in the current literature as either an idiopathic disease or exceedingly rare sequelae after lumbar puncture, spinal trauma, or meningitis. Other studies have more appropriately termed the iatrogenic pathology as a spinal subdural extra-arachnoid hygroma (SSEH), as there is not often a clear cyst wall as in a true arachnoid cyst. However, to the best of our knowledge, none of the previous studies described an SSEH following uncomplicated posterior lumbar surgery, as they have previously involved clear durotomies during the initial operation. Here, we report the case of a 53-year-old woman who presented to the emergency department with a persistent severe orthostatic headache and worsening leg pain, six days following an uneventful L5-S1 discectomy and left L4-5 laminoforaminotomy, without intraoperative durotomy. Lumbar magnetic resonance imaging (MRI) scan revealed a pseudomeningocele and an SSEH extending from the S1 up to the lower thoracic spine, compressing and displacing the cauda equina. Although the hygroma extended up to the lower thoracic spine, surgical exploration was performed only at the index surgical site with bilateral L5 laminectomy, wide durotomy, and wide fenestration of the arachnoid layer. Postoperatively, her headaches dissipated and her pain improved with complete radiographic resolution of the collection. Iatrogenic SSEH is an exceedingly rare complication of posterior lumbar decompression and can occur in the absence of a durotomy during the index surgery. Although these hygromas may span multiple levels, the initial surgical site or the site of known durotomy should be explored first. They can potentially be treated with only a limited durotomy and arachnoid fenestration at a single level rather than at a multilevel arachnoid fenestration.

## Introduction

An intradural spinal arachnoid cyst (ISAC) is a relatively rare entity, the exact incidence of which is still unknown since the majority of them are asymptomatic. Although ISACs are frequently considered to be idiopathic or a result of spinal trauma, arachnoiditis, epidural hematoma, meningitis, or lumbar puncture, which cause either an alteration of cerebrospinal fluid (CSF) flow or direct damage to dura mater, and have been previously documented as a potential etiology of ISACs. More recently, Singleton, et al. [1] appropriately described the iatrogenic entity as a spinal subdural extra-arachnoid hygroma (SSEH), as the CSF was not contained within a complete cyst wall as in a true arachnoid cyst. However, to the best of our knowledge, the formation of SSEHs following spinal surgery has been rarely described in the literature, the majority of which were attributable to intraoperative incidental durotomy [2-6]. Here, we report a case of postoperative formation of an SSEH in a patient who underwent a seemingly uncomplicated posterior lumbar decompression, without intraoperative durotomy, with complete resolution of the cyst following a repair targeted only at the site of the previous operation.

## Case presentation

**Figure 1:**
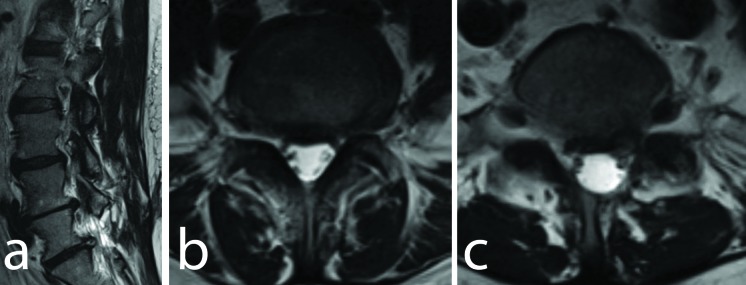
Axial and Sagittal T2W Images (a) Preoperative T2-weighted sagittal magnetic resonance imaging (MRI) scan demonstrating the presence of left L4-5 and L5-S1 disc herniations. (b) Axial T2-weighted MRI at L4-5 level. (c) Axial T2-weighted MRI at L5-S1 level demonstrating foraminal compression but with no evidence of an arachnoid cyst.

**Figure 2:**
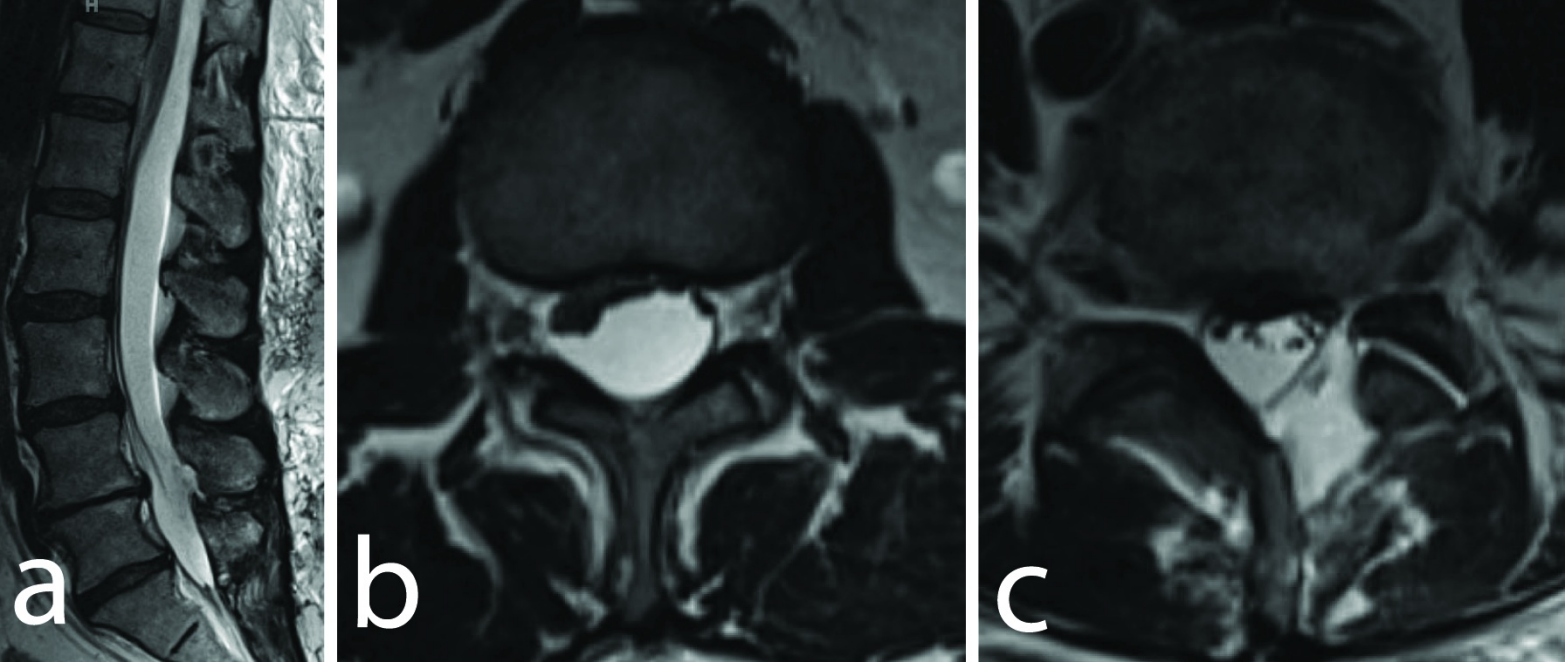
Axial and Sagittal T2W Images (a) Sagittal T2-weighted magnetic resonance imaging (MRI) scan demonstrating the SSEH extending from T12 to S1 and causing compression of the conus medullaris and cauda equina. Axial T2-weighted MRI (b) at the level of the conus and (c) at the L4-5 level demonstrating significant compression and ventral displacement due to the extra-arachnoid hygroma. A small pseudomeningocele is also apparent at the level of the prior hemilaminotomy.

**Figure 3:**
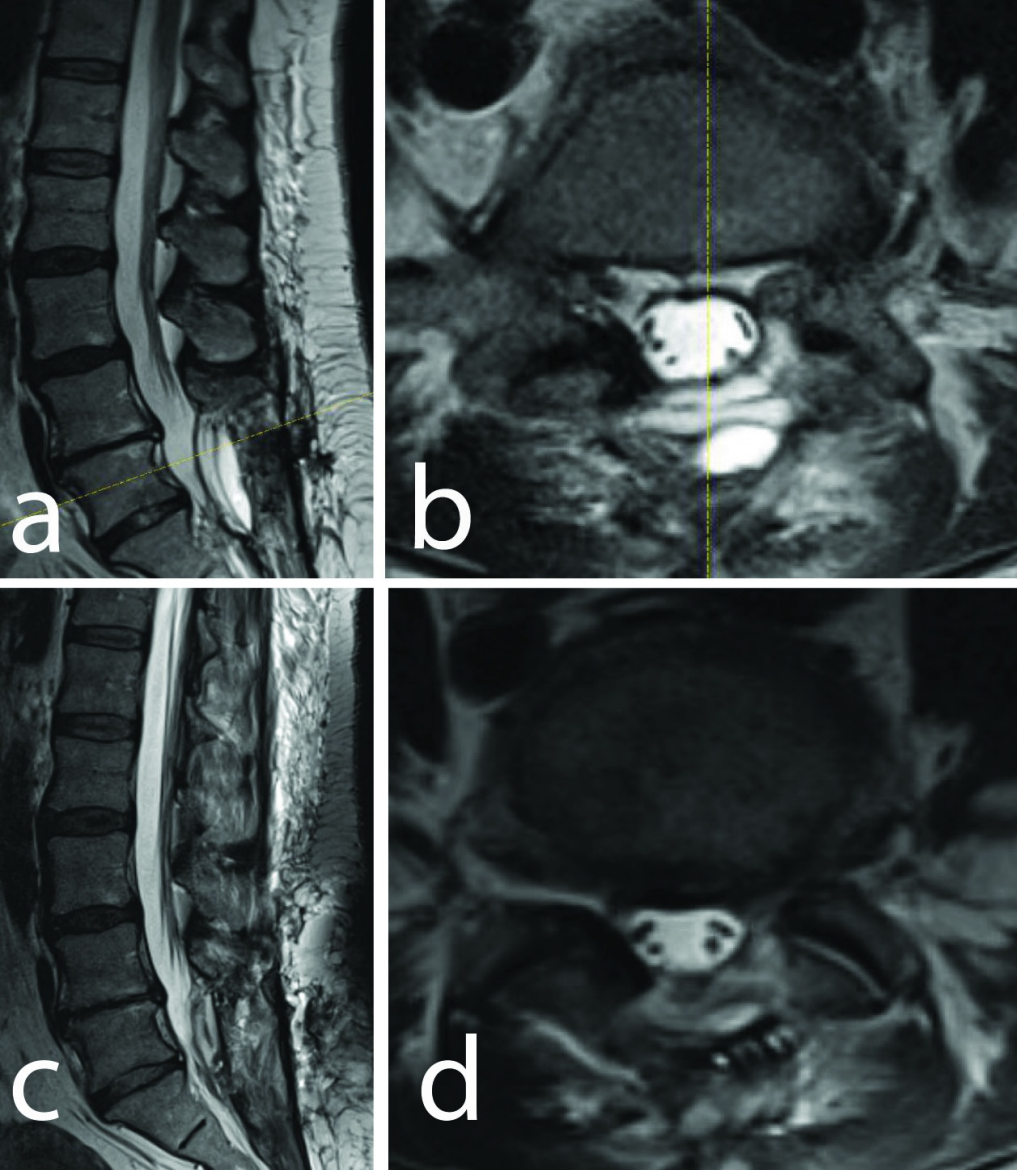
Axial and Sagittal T2W Images (a) Sagittal T2-weighted magnetic resonance imaging (MRI). (b) Axial T2-weighted MRI at the level of L4-5 taken postoperatively demonstrating decompression of the entire SSEH following L5 laminectomy with intradural exploration and arachnoid fenestration. (c) Sagittal T2-weighted MRI (d) Axial T2-weighted MRI at the level of L4-5 taken one month postoperatively demonstrating continued resolution of the entire SSEH.

A 53-year-old woman presented to us with recurrent lumbar radiculopathy, having had a lumbar discectomy elsewhere. She was found to have a recurrent L5/S1 disc herniation and foraminal stenosis at L4/5, with no evidence of intradural arachnoid cyst or hygroma (Figure 1). She underwent a revision left L5-S1 discectomy and left L4-5 laminoforaminotomy, and no intraoperative durotomy was encountered. She was discharged from the hospital routinely. On postoperative day six, she presented to the emergency department with a persistent severe orthostatic headache and worsening leg pain. Lumbar magnetic resonance imaging (MRI) scan demonstrated a sub-fascial fluid collection consistent with pseudomeningocele, which was contiguous with a newly formed SSEH extending from S1 to the lower thoracic spine, causing severe compression and displacement of the cauda equina (Figure 2). She was urgently taken for an L5 total laminectomy where a midline durotomy was created and expanded both cranially and caudally. The arachnoid was identified and fenestrated widely; there was no evidence of a true two-layer arachnoid cyst. The durotomy site was sutured primarily and covered with fibrin glue. A postoperative MRI demonstrated resolution of the SSEH (Figure 2C) and the patient was discharged home in good condition. She continued to do well one month postoperatively, and an MRI performed at that time demonstrated continued resolution of the hygroma (Figure 3).

 

## Discussion

The formation of iatrogenic ISACs following lumbar spinal surgery has been rarely reported in the literature [2-5, 7], and has not been previously reported without an incidental durotomy at the index surgery. For instance, Ford reported five cases of ISACs following lumbar discectomy with clear intraoperative incidental durotomy [7], whereas Nath, et al. reported a case of an anterior cervicothoracic ISAC, 28 years after the initial laminectomy at the same level, in which case the correlation between laminectomy and the ISAC was unclear [5]. Singleton, et al. [1] described two cases in the lumbar spine resulting in cauda equina syndrome, but they more appropriately termed this condition SSEH as there wasn't a true two-layer arachnoid cyst present. However, in contrast to the present case report, they also had durotomies during the index surgeries.

The exact mechanism of this rare complication remains controversial. Some hypothesized that postoperative adhesions surrounding the thecal sac had altered the flow of CSF and subsequently caused an ISAC locally or remotely. In our case, consistent with the theory proposed by Nottmeier, et al. [3], an unrecognized durotomy must have occurred intraoperatively or may have occurred postoperatively (perhaps due to a bone spicule at the decompression site), that led to puncture of the dura and arachnoid. A ball-valve type phenomenon may have occurred at the puncture site, perhaps with intermittent occlusion by a nerve root, leading to the development of the arachnoid cyst [1, 5]. Based on this suspected mechanism, the patient was explored at the initial surgical site only with the performance of an L5 total laminectomy, durotomy, and fenestration of the arachnoid, despite the presence of the hygroma spanning from the lower thoracic spine to the sacrum. The collection then resolved completely.

## Conclusions

Although an SSEH is an exceedingly rare complication of posterior decompression, it should be considered as one of the differential diagnoses (even without intraoperative durotomy noted or appreciated) if a patient presents with progressive neurological complications and orthostatic headache postoperatively. Although these collections may span multiple levels, the initial surgical site or the site of known durotomy should be explored first, as these can potentially be treated with only a limited durotomy and wide arachnoid fenestration, rather than having to perform a multilevel laminectomy.
